# Apoptotic and Cell Cycle Effects of Triterpenes Isolated from *Phoradendron wattii* on Leukemia Cell Lines

**DOI:** 10.3390/molecules27175616

**Published:** 2022-08-31

**Authors:** Lía S. Valencia-Chan, Dafné Moreno-Lorenzana, Jimmy Josué Ceballos-Cruz, Sergio R. Peraza-Sánchez, Antonieta Chávez-González, Rosa E. Moo-Puc

**Affiliations:** 1 Unidad de Investigación Médica Yucatán, Unidad Médica de Alta Especialidad, Centro Médico Ignacio García Téllez, Instituto Mexicano del Seguro Social (IMSS), Calle 41 No. 439, Col. Industrial, Mérida 97200, Mexico; 2 Unidad de Investigación Oncológica, Hospital de Oncología, Centro Médico Nacional, Instituto Mexicano del Seguro Social (IMSS), Avenida Cuauhtémoc 330, Ciudad de Mexico 06720, Mexico; 3 CONACyT–Instituto Nacional de Pediatría (INP), Avenida Insurgentes Sur 1582, Crédito Constructor, Ciudad de Mexico 03940, Mexico; 4 Unidad de Biotecnología, Centro de Investigación Científica de Yucatán (CICY), Calle 43 No. 130, Col. Chuburná de Hidalgo, Mérida 97205, Mexico

**Keywords:** leukemia, lupane-type triterpene, apoptosis, cell cycle, molecular docking

## Abstract

Current antineoplastic agents present multiple disadvantages, driving an ongoing search for new and better compounds. Four lupane-type triterpenes, 3α,24-dihydroxylup-20(29)-en-28-oic acid (**1**), 3α,23-dihydroxy-30-oxo-lup-20(29)-en-28-oic acid (**2**), 3α,23-*O*-isopropylidenyl-3α,23-dihydroxylup-20(29)-en-28-oic acid (**3**), and 3α,23-dihydroxylup-20(29)-en-28-oic acid (**4**), previously isolated from *Phoradendron wattii*, were evaluated on two cell lines of chronic (K562) and acute (HL60) myeloid leukemia. Compounds **1**, **2**, and **4** decreased cell viability and inhibit proliferation, mainly in K562, and exhibited an apoptotic effect from 24 h of treatment. Of particular interest is compound **2**, which caused arrest in active phases (G2/M) of the cell cycle, as shown by in silico study of the CDK1/Cyclin B/Csk2 complex by molecular docking. This compound [3α,23-dihydroxy-30-oxo-lup-20(29)-en-28-oic acid] s a promising candidate for incorporation into cancer treatments and deserves further study.

## 1. Introduction

Worldwide, cancer is the second most common cause of death. Leukemias are among the most frequent types of cancer, and most commonly affect people under 20 years of age [1,2]. Current treatments against leukemia have serious disadvantages as the drugs they involve present multiple side effects, such as limited efficacy versus metastasis and loss of efficacy due to development of resistance mechanisms by cancer cells, among others. Finding new and effective compound is an ongoing challenge [3].

The world’s flora is a seemingly infinite source of bioactive compounds. As part of our search for new efficient and safe alternative agents we have been studying the mistletoe *Phoradendron wattii* (syn.: *Phoradendron vernicosum*), which, in traditional Mayan medicine, is used to treat symptoms suggestive of cancer [4]. We previously isolated four lupane-type triterpenoids from *P. wattii* with favorable characteristics such as good yield, structural stability, biological activity, and selectivity [5].

There are multiple reports of anticancer activities of lupane skeleton triterpenes, mainly betulinic acid, betulin and lupeol, since these compounds are ubiquitous in most plants [6], as well as 23-hydroxybetulinic acid [7]. The selective inhibitory activity of betulic acid was of interest for the study of other lupane skeleton triterpenes, as well as to determine their mode of action, and their ability to affect other cancer cell lines, such as leukemic cells; thus showing that the main mechanism of death is by cell apoptosis, such as the loss of potential of the mitochondrial membranes (MMP), DNA fragmentation [8], upregulation of the expression of the pro-apoptotic protein Bax [9], release of both cytochrome c and Smac [10], release of TNFα, stimulation of TNFR, activation of caspases, production of reactive oxygen substances (ROS) and consumption of glutathione (GSH) with accumulation of its oxidized form (GSSG) [11], in addition to the arrest of the G1/S phase of the cell cycle [7].

These triterpenoids’ properties, along with previous reports on pentacyclic triterpenes’ action against leukemia cells [7,12], suggested to us the need to assess their effects in chronic and acute myeloid leukemia cells. The present study objective was to evaluate the effect of these metabolites against the K562 and HL60 myeloid leukemia cell lines.

## 2. Results

**Figure 1:**
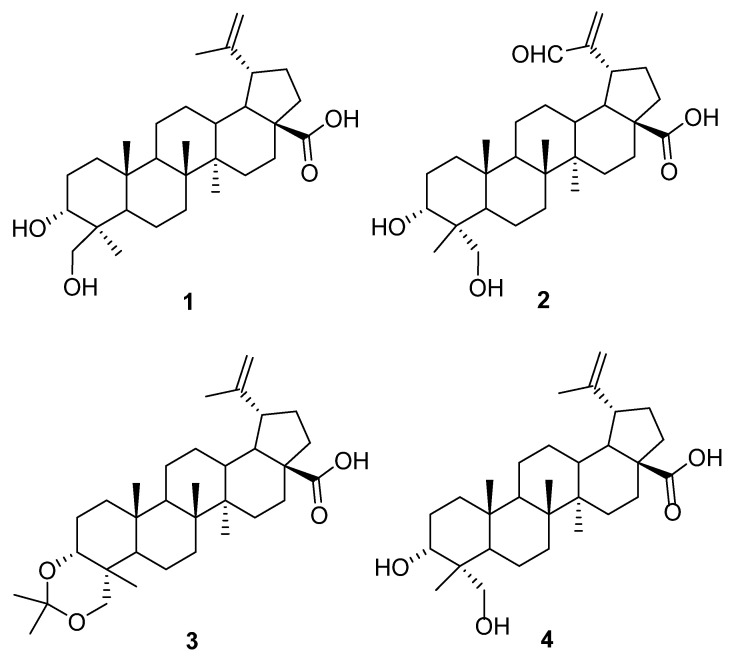
Chemical structures of compounds **1**–**4**.

**Figure 2:**
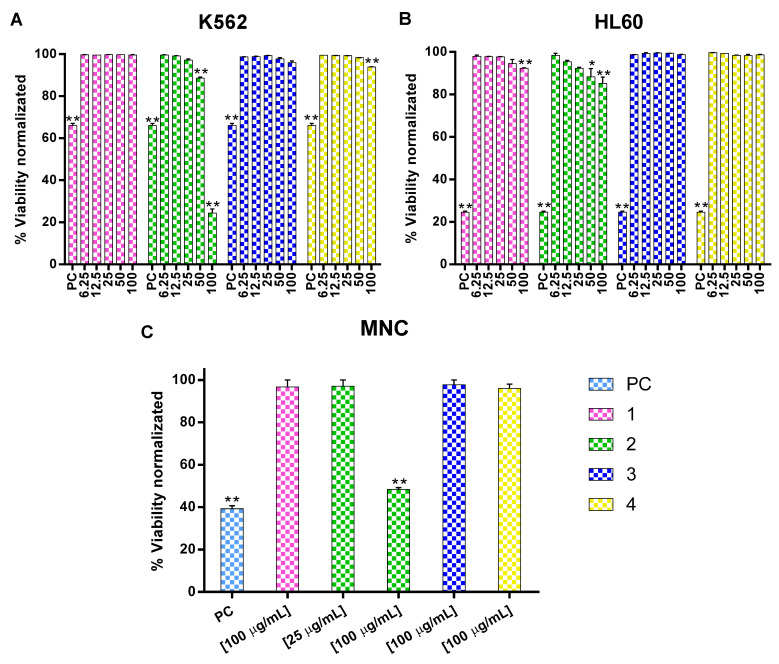
Effect of compounds **1**–**4** at different concentrations in K562 (**A**) and HL60 (**B**) cell and normal MNC (**C**). Normal and leukemia cells were cultured in presence of different compounds concentration by 48 h and the viability percentage was analyze using 7-AAD. Data are expressed as the percentage of 7-AAD ± SEM positive cells from at least three different experiments in triplicate. Statistical significance was determined by a one-way analysis of variance (ANOVA) followed by *Dunnett’s post-hoc test*. The differences were considered significant * *p* < 0.05, ** *p* < 0.01 vs. negative control (NC).

The effect on leukemia cells viability was evaluated using different concentrations of four isolated lupane-type triterpenes: 3α,24-dihydroxylup-20(29)-en-28-oic acid (**1**); 3α,23-dihydroxy-30-oxo-lup-20(29)-en-28-oic acid (**2**); 3α,23-*O*-isopropylidenyl-3α,23-dihydroxylup-20(29)-en-28-oic acid (**3**); and 3α,23-dihydroxylup-20(29)-en-28-oic acid (**4**) (Figure 1). In the K562 cell line, compound **2** was capable of reducing cell viability to 88.6 ± 0.40% at 50 µg/mL and to 24.3 ± 1.95% at 100 µg/mL (Figure 2A). In the HL60 cell line, compound **1** reduced cell viability to 92.25 ± 0.25% at 100 µg/mL, a significant difference compared to the negative control. In the same cell line, compound **2** decreased cell viability to 88.35 ± 3.85% at 50 µg/mL and 85.15 ± 3.05% at 100 µg/mL (Figure 2B).

Compound safe was measured by evaluating them against normal mononuclear cells (MNC). Only compound **2** at 100 µg/mL reduced cell viability (48.3 ± 0.88%), whereas the other compounds did not differ from the negative control (Figure 2C).

Their antiproliferative effect was evaluated at different concentrations in vitro. Cells were pre-stained with carboxyfluorescein succinimidyl ester (CFSE) and, for 48 h, were placed in the presence or absence of triterpenes at a specific concentration, as well as a positive control (PC). After this time, they were stained with 7-aminoactinomycin D (7-AAD) to select surviving cells and further analyses by cytometry flow (Figure S1).

**Figure 3:**
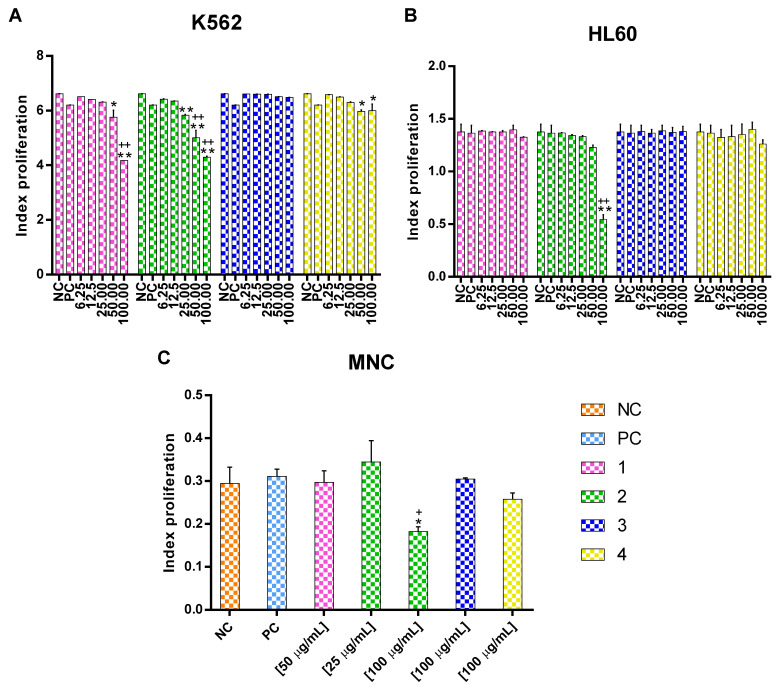
The data represent mean ± SEM of proliferation index from three different experiments K562 (**A**), HL60 (**B**) cell lines and normal MNC (**C**) at different concentrations. * *p* < 0.05 and ** *p* < 0.01 were compared to the negative control. ^+^ *p* < 0.05 and ^++^ *p* < 0.01 were compared to the positive control. Statistical significance was determined by a one-way analysis of variance (ANOVA) followed by *Dunnett’s post-hoc test*.

To facilitate comparison with the control when using the K562 cell line, the index of proliferation (IP) was considered as zero (T0) at the beginning of culture. After 48 h, the cells exposed to compound **1** exhibited an IP of 5.76 ± 0.26 at 50 μg/mL and 4.18 ± 0.01 at 100 μg/mL. In the treatment with compound **2**, the IP was 5.83 ± 0.30 at 25 μg/mL, 5.01 ± 0.27 at 50 μg/mL, and 4.29 ± 0.03 at 100 μg/mL. Finally, in the treatment with compound **4** the IP was 5.97 ± 0.06 at 50 μg/mL and 6.00 ± 0.24 at 100 μg/mL. Compounds **1**, **2**, and **4** had a concentration-dependent effect on delaying cell proliferation (Figure 3A and Figure S1A). With the HL60 cell line, only compound **2** delayed proliferation, with an IP 0.54 ± 0.45% at 100 μg/mL (Figure 3B and Figure S1B). Although compounds **1**, **2**, and **4** delayed leukemia cell proliferation, compound **2** was clearly the most effective. Even so, the chronic myeloid leukemia (CML) cells were more susceptible to it than the acute myeloid leukemia (AML) cells.

When all compounds were evaluated on MNC at the concentrations for which the response differed from the negative control in the two leukemia cell lines Figure 3C and Figure S1C), no difference was observed with compounds **1**, **3**, and **4**. However, at 100 μg/mL compound **2** exhibited an IP of 0.18 ± 0.11. Worth noting is that at this same concentration compound **2** caused a greater reduction in the CFSE level in the K562 cell line, implying a higher potency against this CML cell line and notable selectivity.

**Figure 4:**
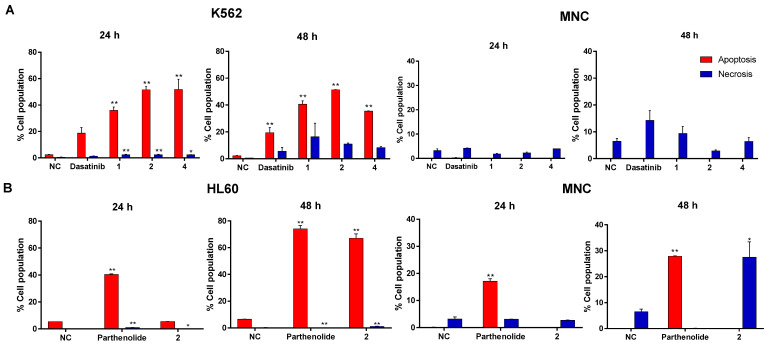
The compounds **1**, **2**, and **4** induced apoptosis in K562 and compound **2** in HL60. The compounds **1** (50 µg/mL), **2** (25 µg/mL), and **4** (100 µg/mL) were evaluated in cell line K562 and normal MNC. The results in (**A**) represents mean ± SEM of at least three independent experiments in cell line K562 and normal MNC, and results in (**B**) represents mean ± SEM of at least three independent experiments in cell line HL60 and normal MNC of compound **2** (100 µg/mL). Statistical significance was determined by a one-way analysis of variance (ANOVA) followed by *Dunnett’s post-hoc test*. The differences were considered significant at * *p* < 0.05, ** *p* < 0.01 compared to negative control.

A double stain was used to quantify the cell death rate from the apoptosis (annexin V-FITC positive) or necrosis (single 7-AAD positive) pathways at 24 and 48 h. At 24 h with the K562 cell line, death by apoptosis was 2.27 ± 0.31% in the negative control (Figure 4A and Figure S2A, marked in red box), while in the positive control (dasatinib), it increased to 18.72 ± 4.2%. Death by apoptosis was clearly higher in the three active compounds: 35.95 ± 2.55% (**1**), 51.50 ± 2.65% (**2**), and 51.80 ± 7.80% (**4**). After 48 h, no change had occurred in the negative (2.07 ± 0.26%) and positive (19.34 ± 3.86%) controls, although with compound **1** death by apoptosis increased to 40.60 ± 2.50%. No change was observed with compound **2** at this time (51.33 ± 0.22%), and with compound **4** the rate of apoptosis was lower (35.45 ± 0.25%) than at 24 h. When the compounds were evaluated under the same conditions on MNC, most cells remained viable (annexin V-FITC-, 7-AAD-), and only a small proportion were necrotic (annexin V-FITC-, 7AAD+). These results highlight triterpenes **1**, **2**, and **4** had minimal effect on normal cells, but clearly induced apoptotic death in leukemia cells to varying degrees.

When tested against the HL60 cell line (Figure 4B and Figure S2B) at 24 h the negative control exhibited 5.36 ± 0.20% apoptosis, and the positive control (parthenolide) 40.40 ± 0.50%. After 48 h, the rate had changed little in the negative control (5.54 ± 0.40%), but had increased in the positive control (74.23 ± 2.37%). Of the tested compounds, only **2** at 100 µg/mL exhibited an effect: 5.54 ± 0.40% apoptosis at 24 h and 67.08 ± 3.31% at 48 h. The HL60 line is clearly less sensitive to this compound than the K562 line.

Under the same conditions, the apoptosis rate in normal MNC exposed to compound **2** for 24 h was minimal, but at 48 h, necrosis had reached 35.23 ± 2.20% (annexin V-FITC-, 7AAD+). 

To identify cell cycle status, we used flow cytometry to evaluate distribution of the different cell cycle phases for each compound in the two leukemia cell lines at 48 h. 

**Figure 5:**
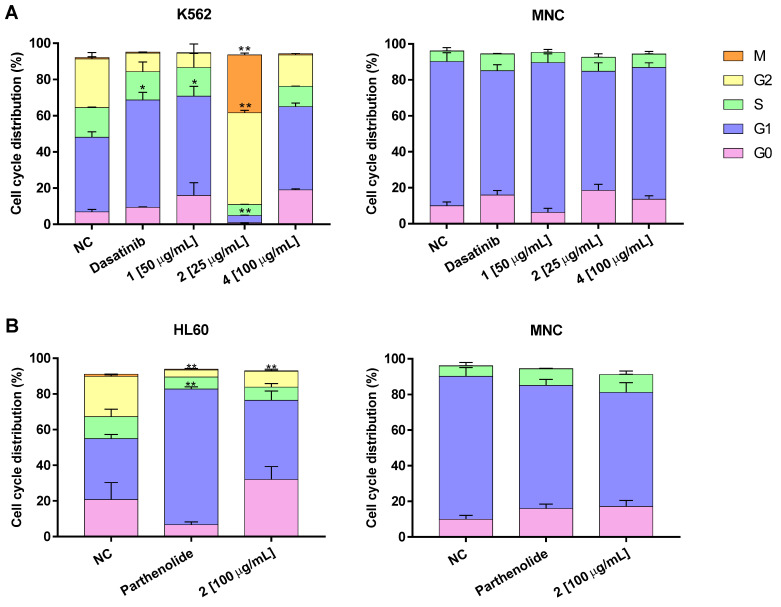
Results in cells K562 (**A**) and HL60 (**B**) of different compounds on the cell cycle status, represent mean ± SEM from three different experiments of cell cycle phase distribution in the different cells analyzed. Significance between cell cycle phase was determined using one-way analysis of variance (ANOVA) followed by *Dunnett’s post-hoc test*. Differences were considered significant at * *p* < 0.05, ** *p*< 0.01 vs. negative control (NC).

In the negative control, the K562 line had 41.2 ± 3.0% of cells in G1 phase, 26.9 ± 3.4% in G2, 16.35 ± 0.01% in G0, and 10.2 ± 0.2% in S (Figure 5A and Figure S3A). In the positive control (dasatinib), cells in G0 accounted for 59.3 ± 4.2% of the total and G1 for 10.2 ± 0.2%. With compound **1** the G0/G1 was 70.8% and with compound **4** it was 65.0%, comparable to the positive control. No notable G0/G1 accumulation was observed with compound **2**; G1 accounted for 3.92% and the active cell cycle phases (G2/M) for 82.6%. None of the tested compounds exhibited any effect on MNC since cell distribution differed minimally between the culture conditions. This is relevant because elimination of the G0 cell cycle phase or induction of cell cycle transit to active phases has not been reported for dasatinib and imatinib, the drugs of choice in leukemia treatment.

In the HL60 cell line, compound **2** raised the G0/G1 phases to 83.0%, which does not differ from the 74.4% with the positive control, parthenolide (Figure 5B and Figure S3B). Compound **2**′s effect on normal MNC cells did not differ from either the positive or negative controls.

Given the above results, it was decided to do molecular docking analysis using key targets reported for leukemia cells; docking scores for compounds **1**–**4** were compared to ATP and a co-crystalized inhibitor (Table 1). All four compounds’ binding energies for the VEGFR (Vascular Endothelial Growth Factor Receptor) kinase domain were higher than the original substrate adenosine triphosphate (ATP) and the co-crystalized inhibitor (tivozanib). This indicates that the observed effects of compounds **1**–**4** on K562 and HL60 cells are not related to this target (Figure S1). However, a correlation was observed with the FLT-3 (FMS-like tyrosine kinase 3) protein in the HL60 cell line, compound **2** showing a lower score than compounds **1**, **3**, and **4** (Figure S4).

**Figure 6:**
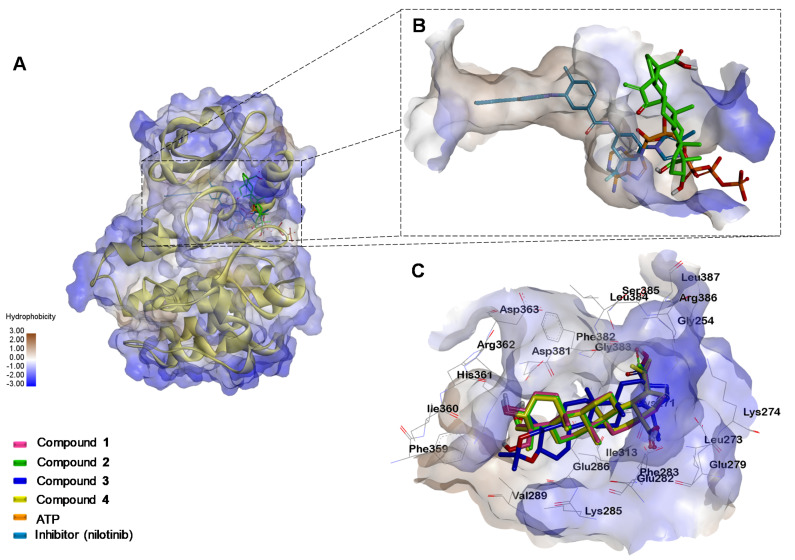
Binding modes of the compounds under study at the BCR-ABL binding site. (**A**) Binding site of nilotinib, ATP and compound **2**. (**B**) Close-up of the binding site of nilotinib (Re-docking RMSD: 0.717 Å), ATP and compound **2**. (**C**) Superimposition of compound **1**, **2**, **3**, and **4** into ATP binding site of ABL kinase.

**Figure 7:**
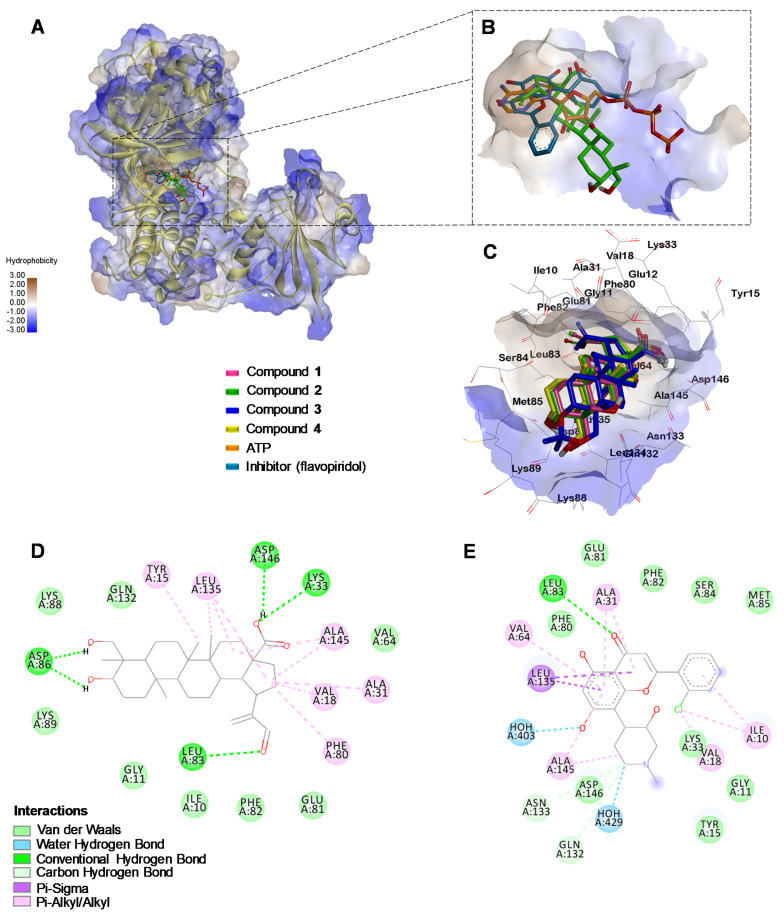
(**A**) Binding modes of the compounds under study into CDK1/Cyclin B/Csk2 binding site. (**B**) Close-up of the binding site of flavopiridol (Re-docking RMSD: 0.462 Å), ATP and compound **2**. (**C**) Visualization of interactions between compounds **1**–**4** inside the binding site of CDK1. (**D**) Visualization of interactions between compound **2** inside the binding site of CDK1. (**E**) Visualization of interactions between flavopiridol inside the binding site of CDK1 in raw complex retrieved from PDB.

In contrast, for ABL kinase (Figure 6) and the CDK1/CyclinB/Cks2 complex (Figure 7) the scores for compounds **1**–**4** with experimental bioactivity are better correlated; in which compound **3** had the highest score, epimers **1** and **4** had comparable scores, and compound **2** had the lowest. The high scores suggest involvement of these two targets in the effects observed during the experimental analysis. 

As a complementary study, we submitted the four compounds’ molecular structures to in silico analysis via the SwissADME platform to predict their physicochemical and pharmacokinetic properties (Table 2).

Lipinski’s rule of five for compound selection was applied as an initial approach. Only compound **2** complied with all the rules, with acceptable values for molecular weight (≤500), hydrogen bond donors (nHBA ≤ 5), hydrogen bond acceptors (nHBD ≤ 10), lipophilicity (expressed in cLogP ≤ 5), molar refractivity (TPSA ≤ 140 Å) and rotatable bonds (nROTB ≤ 10) [13]. It was predicted that compound **2** would have high passive gastrointestinal absorption and would not inhibit some key CYP450 isoforms (Table 2), making it a good candidate for further study [14].

## 3. Discussion

The observed in vitro effect of triterpenes **1**–**4** on leukemic cells suggests that some functional groups in these molecules are prone to interact with active sites such as the hydroxyl groups, thus improving their anti-cancer activity. For example, when the hydroxyl groups at C-3 and C-4 are protected with an acetonide, these triterpenes essentially have no activity (CI_50_ > 100 µg/mL).

Compounds **1** and **4** are epimers, these two molecules differ in their C-4 and thus in the orientation of the hydroxymethyl group, either forward (C-24) or backward (C-23), respectively. Compound **1** had a more substantial antiproliferative effect against the K562 cell line than compound **4**. Based on these differences in activities and stereochemistry, interaction with enzymes or receptors is the main possible mechanism of action. 

Of all the evaluated compounds, compound **2** exhibited the most notable effect against leukemia cells. This may be due to the presence of the formyl group at C-30, in the form of α,β-unsaturated aldehyde, which has increased activity against leukemia cells compared to a non-functionalized analog [15]. This oxidized group apparently induces a more selective effect against leukemia cell lines than against solid tumors [16]. Previous research has shown presence of the aldehyde group on C-28 in lupane-type triterpenes to exhibit preferential against leukemia cell lines compared to that exercised against solid tumors and normal cells. This has also been observed in other compounds with other functionalization which induce cell death by apoptosis [17]. Triterpenes with a lupane-type skeleton and an α,β-unsaturated aldehyde group, specifically at C-30, exhibit similar increasing activities in adherent cell lines due to the presence of this functionalization, with a further increase due to simultaneous oxidation at C-28 [18,19].

One of the mechanisms by which the presence of α,β-unsaturated carbonyl groups (e.g., ketones or aldehydes) seems to induce a cytotoxic effect is through generation of Michael-type adducts. This is due to the reactivity of the β-carbon with the nucleophilic thiol group of proteins and peptides, which play key role in cancer progression and drug resistance. In addition to their ability to interact with cellular thiols, compounds with α,β-unsaturated carbonyl groups can induce apoptosis via the mitochondrial pathway, which is considered one of the main physiological cell death pathways and a promising therapeutic target for cancer treatment [20,21].

Worth noting is that the percentage of leukemia cells arrested in the inactive phases of the cell cycle (G0/G1) after in vitro treatment with compound **2** was lower compared to that shown by drugs currently used to treat chronic myeloid leukemia, such as tyrosine kinase inhibitors (TKIs) (e.g., imatinib or dasatinib). The mechanism of TKIs on the cell cycle is to arrest cells in inactive phases, causing them to remain in a resting state, making their elimination (and death) difficult and resulting in persistent post-treatment disease [22,23,24]. Unfortunately, most patients treated with TKs, such as dasatinib and imatinib, experience leukemia cell persistence in CML because the leukemia stem cells (CD34 + CD38-) enter the G0 phase, in which the cells become quiescent, and can thus survive.

The fact that compound **2** caused arrest in the active phases of the cell cycle suggest that it may be affecting the CDK1 responsible for cell cycle regulation in the G2 and M phases [25]. Known to be overexpressed in many types of cancer, CDKs have become attractive therapeutic targets for preventing unregulated cancer cell proliferation [26]. This makes compound **2** a promising candidate in future studies, mainly aimed at CML, because it exhibits interactions similar to those of flavopiridol, coupled with the fact that it permits MNC cells to remain highly viable.

Given the observed behavior of compound **2** against leukemia cells, a combination of compound **2** with TKI drugs, such as imatinib, is a promising approach. Combinations of TKIs with drugs that inhibit active phases of the cell cycle, such as vinca alkaloids [27] or paclitaxel, have been used to treat advanced or metastatic solid tumors [28]. 

Compound **2** was the only one effective on the HL60 cell line. This is promising, since there is currently no effective treatment for CML or AML. The FLT3 protein is known to one of the targets in AML. This type III tyrosine kinase receptor plays vital role in the survival, proliferation and differentiation of hematopoietic cells in AML [29]. In the present molecular docking results with FLT3, compound **2** had a better score that compounds **1**, **3**, and **4**, suggesting that interaction with FLT3 may be involved in the activity observed in the HL60 cell line; however, the scores were lower for the inhibitor and ATP with the same protein.

The VEGFR was selected as a molecular docking target because it plays key role in the proliferation and survival of leukemic cells [30,31]. In the present results none of the effects exerted by compounds **1**–**4** were found to be linked to interaction with this target.

When tested with BCR-ABL protein, one of the most potent anti-apoptotic proteins in CML [32], compounds **1**–**4** could not bind in the same central cavity as this inhibitor (also occupied by the nucleoside portion of ATP). However, they could bind in the larger cavity constituting the “entrance” to the central cavity, thus hindering entry of ATP into the site of action, and consequently, inhibiting the kinase activity (Figure 6C). According to the docking scores, compound **2**, and to a lesser extent epimers **1** and **4**, are capable of competing with ATP; this suggests that the observed apoptotic effect is due to this interference with the ABL signaling pathway, which in turn can trigger cell death in K562 cell line.

The molecular docking analysis using CDK1 showed all four triterpenes to be capable of entering the same site as the competing inhibitor, with a similar position and orientation (Figure 7B). However, compound **2** had the lowest score of all the tested triterpenes, even lower than ATP, and one comparable to the inhibitor flavopiridol. This low score may have resulted from formation of five interactions through hydrogen bonds with residues ASP86, ASP146, LYS33, and LEU83 (Figure 7C); the latter may be a key interaction since it also occurs with flavopiridol (Figure 7D), but not with the other triterpenes (Figure S3). Based on these remarkable results for interaction with CDK1, the principal effect observed for compounds **1**–**4** may be explained by their interaction with the CDK1/Cyclin B/Cks2 complex, an important mitotic promoter [25]. It may also account for the significant arrest in the G2/M phases caused by compound **2**, which is one of flavopiridol’s modes of action.

The predicted properties of analysis indicated compound **2** to be the most promising of the four compounds since it is the only one which meets all the parameters of Lipinski’s rule of five. These criteria are aimed at assisting in development of orally bioavailable drugs with high gastrointestinal absorption, a vital goal in drug discovery. The fact that compound **2** was not predicted to be a CYP450 isoenzymes inhibitor suggests it has a lower possibility of being toxic and/or it may lessen the appearance of undesirable side effects such as elimination and accumulation of drugs and their byproducts [14,33,34,35].

As observed, compound **2** has an important effect on leukemic lines; however, like other triterpenes, they show a significant drawback due to their moderate solubility (SwissADME), presumably related to their lipophilicity, since it is well known that the low aqueous solubility of a drug can seriously affect its efficacy and hinder its possible therapeutic application in clinical use as a medicine [36,37,38].

A large number of studies have been carried out to improve their pharmacological activities of triterpenes, such as by introducing heterocyclic scaffolds, mainly nitrogen-containing heterocyclics, such as triazole, pyrazole, indole, piperazine and aminoquinolines [38], as well as the preparation of esters, amides, saponins or conjugated sugars [36,39], esters with dicarboxylic acids, conjugated with polyethylene glycol, ammonium salts, among others [40].

Another way to improve bioavailability is by encapsulating them in nanoparticles, such as liposomes, which have been shown to be efficient and non-toxic, capable of incorporating the triterpene scaffold, such as betulinic acid, through the use of relatively inexpensive materials and manufacturing procedures [37].

The implementation of delocalized lipophilic cationic compounds such as Rho123, F16, MKT-077, qualinium and triphenylphosphonium lipophilic cationic salts to pentacyclic triterpenes has also been explored [41], the latter being of great importance, since in addition to improving water solubility, and thus bioavailability, targets mitochondria [41].

The pentacyclic triterpenes, such as betulin and betulinic acid; have apoptotic effects on cancer cells through initiating the formation of ROS and free radicals on mitochondria, increasing the permeability of the mitochondrial membrane, which results in the release of cytochrome c in the cytosol and activation of caspases to end DNA fragmentation. These compounds also have effects on the Bcl-2 protein family, which consists of anti-apoptotic (Bcl-2, Bcl-XL and Mcl-1) and proapoptotic (Bax and Bad) proteins [41,42,43].

However, due to low bioavailability, it is difficult *in vivo* conditions to produce the desired therapeutic effect. Therefore, binding to lipophilic cationic compounds would improve their activity, mainly by inducing apoptosis directly in the effect on mitochondria in cancer cell lines [41,42,43].

However, compound **2**, despite showing moderate solubility and a LogP of 4.61, is better than its analogs that have a LogP of up to 6.65 and poor solubility in water (Compound **3**). What this leads us to make modifications or formulations in the future to improve its pharmacokinetic parameters, because compound **2** shows promise for future studies [37].

## 4. Materials and Methods

### 4.1. Compounds

The compounds were isolated from the aerial parts of *Phoradendron wattii* according to a previous study [5]. Compounds were dissolved in DMSO at a concentration of 10 mg/mL.

### 4.2. Controls

In all cases medium with DMSO (0.1%) was used as negative control, dasatinib (2 nM) and parthenolide (10 µM) were used as positive controls for K562 and HL60 cell lines, respectively. All tests were carried out in triplicate.

### 4.3. General Procedures

The bioassays were performed inside a laminar flow hood, brand NuAire, class II, type A2. The cells were kept inside a CO_2_ incubator with a water jacket and HEPA filter, brand NuAire.

### 4.4. Cell Lines

Chronic myeloid leukemia (K562; ATCC CCL-243) and acute myeloid leukemia (HL60; ATCC CCL-240) cell lines were used for all assays. The K562 cell line was maintained with RPMI medium at 10% FBS, the HL60 cell line was maintained with IMDM medium at 20% FBS; 1% penicillin-streptomycin was added to both cell lines and incubated at an atmosphere of culture with 95% humidity and 5% CO_2_ at 37 °C. All evaluation was performed between 4 and 5 cell passages.

### 4.5. Bioassay of Viability

A total of 2 × 10^5^ cells/well were cultured into 24-well plates and treated with different concentrations of four compounds (6.25, 12.5, 25, 50, and 100 μg/mL) for 48 h. At the end of this time, the cells were collected and stained with 7-aminoactinomycin D (7-AAD), according to manufacturer instructions, for 15 min in darkness. The samples were then analyzed by flow cytometry using FACS Verse flow cytometer (BD Bioscience, San Jose, CA, USA).

### 4.6. Viability Test in Normal Mononuclear Cells

Normal mononuclear cells were obtained from bone marrow samples, and were collected following institutional guidelines, including written informed consent from each donor. Collection procedures were approved by the Ethics and Scientific Committees of the Mexican Institute of Social Security [Instituto Mexicano del Seguro Social (IMSS), project R-2013-3602-6].

The MNC were purified with FicollPaque Plus (Pharmacia Biotech, Uppsala, Sweden) by centrifugation at 400× *g* at room temperature for 30 min according to the manufacturer’s protocol. Once the MNC were obtained, they were resuspended in RPMI medium (ATCC) with 10% FBS, and they were counted using a hemocytometer, previously stained with a trypan blue solution, verifying the viability of 95% [44].

Cells were plated at 2 × 10^5^ cells/well in 24-well plates and incubated with different concentrations of compounds **1** (50 µg/mL), **2** (25 and 100 µg/mL), **3** (100 µg/mL), and **4** (100 µg/mL), for 48 h. RPMI with 10% FBS was used as culture medium for cell growth. After this time, cells were collected, washed with PBS and stained with 7-AAD, according to manufacturer instructions. They were analyzed by flow cytometry with a FACSVerse flow cytometer (BD Bioscience, San Jose, CA, USA).

### 4.7. Proliferation Bioassay

Cell lines were stained with carboxyfluorescein diacetate succinimidyl ester (CFSE) at 1 mM and incubated at 37 °C for 15 min under constant agitation, to then be washed with PBS at 10% of FBS to eliminate the excess of the dye. 2 × 10^5^ cells/well were seeded in 24-well plates and incubated with different concentrations of the compounds (6.25, 12.5, 25, 50, and 100 μg/mL) for 48 h. After culturing, cells were stained with 7-AAD and immediately analyzed using a FACS Verse flow cytometer (BD Bioscience, San Jose, CA, USA).

### 4.8. Apoptosis Assay

Cells (2 × 10^5^) were cultured in 24-well plates and treated with the compounds at the concentration in which the compounds showed inhibition of viability or proliferation [**1** (50 µg/mL), **2** (25 and 100 µg/mL), **3** (100 µg/mL) and **4** (100 µg/mL)], to later be incubated for 24 h and 48 h. After this time, cells were washed with PBS and stained with annexin V-FITC and 7-AAD apoptosis kit (BD Bioscience) and incubated in the dark for 15 min, then they were analyzed by flow cytometry.

### 4.9. Cell Cycle Assay

Two million cells of K562, HL60, and normal MNC were cultured in 6-well plates and incubated for 48 h with the compounds at different concentrations. After this time, the cells were collected, purified with Ficoll Paque Plus, washed with PBS, and fixed with 4% formaldehyde for 15 min on ice. After that, cells were permeabilized for 20 min with 0.1% triton. Next, cells were washed with PBS 3% FBS and then stained with anti Ki67 antibody (AF488 in 1:100 dilution) for 12 h, washed with PBS 3% FBS and subsequently incubated with 4′,6-diamidino-2-phenylindole dihydrochloride (DAPI) at 500 ng/mL DAPI for 30 min. After this procedure, cells were analyzed using a FACS Canto II flow cytometer (BD Bioscience, San Jose, CA, USA).

### 4.10. In Silico Studies

The ADME (absorption, distribution, metabolism, and excretion) properties of compounds, such as molecular weight (MW), hydrogen bond acceptors (nHBA), hydrogen bond donors (nHBD), lipophilicity (clog*P*), rotatable bonds and topological polar surface area (TPSA), and pharmacokinetic parameters were predicted using SwissADME [14].

Protein target structures for molecular docking studies were retrieved from RCSB Protein Data Bank, selecting CDK1/CyclinB/Cks2 complex with flavopiridol as CDK1 inhibitor (PDB ID: 6GU2), ABL kinase with nilotinib as inhibitor (PDB ID: 3CS9), FMS-like tyrosine kinase 3 (FTL3) with quizartinib as inhibitor (PDB ID: 4RT7) and VEGFR2 kinase domain with tivozanib as inhibitor (PDB ID: 4ASE) [45]. Three-dimensional structures of tested ligands were generated in their low-energy conformation using ChemDraw Professional v.16, based on crystallographic data of the compound or a closely related structure when available, and further energy minimization with MM2 force field. Targets were prepared by removing co-crystalized inhibitors and water molecules, and then processed by LePro tool. Molecular docking studies were performed using LeDock software [46]. A receptor grid was set for each target, restraining it to include position of co-crystalized inhibitor and surrounding cavities in accordance with previous reports for new inhibitor search [47,48,49,50,51,52,53] (resulting search volumes shown in Table 3), generated poses were set to 100 and 0.5 of RMSD. Generated poses and interactions were analyzed in BIOVIA Discovery Studio Visualizer v19.1.

### 4.11. Analysis of Results

All cytometric data was analyzed using FlowJo^TM^ v10.6 software. The data were expressed as means ± SEM. Statistical significance was calculated with a one-way analysis of variance (ANOVA) followed by *Dunnett’s post-hoc* test, applying a *p* < 0.05 significance level.

## 5. Conclusions

The epimers 3α,24-dihydroxylup-20(29)-en-28-oic acid (**1**) and 3α,23-dihydroxylup-20(29)-en-28-oic acid (**4**) and compound 3α,23-dihydroxy-30-oxo-lup-20(29)-en-28-oic acid (**2**) induced inhibition of proliferation and cell death by apoptosis. Molecular docking results suggest this may be due to interaction with the ABL kinase protein. Compound **2**, in particular, allows MNC to remain highly viable. The compound 3α,23-dihydroxy-30-oxo-lup-20(29)-en-28-oic acid (**2**) is promising because it exhibited notable activity in both cell lines, arrested cells in the active phases of the cell cycle, and it was found, in silico, to interact with CDK1/Cyclin B/Csk2 complex. Productive future research should evaluate compound **2**′s effects in leukemia stem cells, as well as implement in vivo bioavailability and toxicity studies.

## Figures and Tables

**Table 1:** Docking scores for compounds **1**–**4**.

Compound	Score (kcal/mol)			
	VEGFR2 Kinase Domain	FLT3	ABL Kinase	CDK1-CyclinB
**1**	−5.20	−5.22	−6.14	−5.12
**2**	−4.89	−6.23	−7.12	−6.48
**3**	−4.62	−5.54	−4.92	−4.72
**4**	−5.16	−5.84	−6.16	−5.08
ATP	−8.33	−7.65	−7.41	−6.44
co-crystalized inhibitor	−10.23	−10.54	−11.46	−6.93

**Table 2:** In silico predicted physicochemical and pharmacokinetic parameters of compounds **1**–**4**.

		Compound			
		1	2	3	4
**Molecular Weight (g/mol)**		472.70	486.68	512.76	472.70
Physicochemical Parameters	nHBA	4	5	4	4
	nHBD	3	3	1	3
	cLogP	5.52	4.69	6.65	5.45
	nROTB	3	4	2	3
	TPSA (Å^2^)	77.76	94.83	55.76	77.76
Pharmacokinetic Parameters	GI absorption	High	High	Low	High
	CYP1A2 inhibitor	No	No	No	No
	CYP2C19 inhibitor	No	No	No	No
	CYP2C9 inhibitor	Yes	No	No	Yes
	CYP2D6 inhibitor	No	No	No	No
	CYP3A4 inhibitor	No	No	No	No

nHBA: hydrogen bond acceptors, nHBD: hydrogen bond donors, clog*P*: lipophilicity (consensus Log *P*_o/w_), nROTB: rotatable bonds, TPSA: topological polar surface area; GI absorption: Gastrointestinal absorption; CYP1A2: cytochrome P450 1A2; CYP2C19: cytochrome P450 2C19; CYP2C9: cytochrome P450 2C9; CYP2D6: cytochrome P450 2D6; CYP3A4: cytochrome P450 3A4.

**Table 3:** Search volume for molecular docking.

PDB Entry	Search Volume					
	Minimum (Å)			Maximun (Å)		
	x	y	z	x	y	z
6GU2	318.71370	205.75305	181.98100	336.44230	228.73095	201.80300
3CS9	14.46000	−5.72656	44.42565	45.40620	21.22764	63.53435
FLT3	−50.71070	−3.84690	−29.64010	−22.42360	24.18290	3.17570
4ASE	−42.91900	−19.71083	−23.08805	−8.47920	9.87587	1.26045

## Data Availability

Not applicable.

## Supplementary Materials

The following supporting information can be accessed at: https://www.mdpi.com/article/10.3390/molecules27175616/s1, Figure S1. Binding modes of the studied compounds at the VEGFR binding site. (A) Binding site of tivozanib, ATP and compound **2**, (B) Close-up of the binding site of tivozanib (Re-docking: 0.286 Å), ATP and compound **2**; Figure S2. Binding modes of studied compounds at the FLT3 binding site. (A) Binding site of quizartinib, ATP and compound **2**, (B) Close-up of the binding site of quizartinib (Re-docking: 1.472 Å), ATP and compound **2**; Figure S3. Interactions of the compounds **1**, **3**, and **4** at CDK1 binding site. (A) visualization of interactions between compound **1** inside CDK1 binding site, (B) Visualization of interactions between compound **3** inside CDK1 binding site, (C) Visualization of interactions between compound **4** inside CDK1 binding site; Figure S4. Binding modes of the studied compounds at the VEGFR binding site; Figure S5. Binding modes of studied compounds at the FLT3 binding site; Figure S6. Interactions of the compounds 1, 3, and 4 at CDK1 binding site.

## References

[1] González-Salas W.M., Olarte-Carrillo I., Gutiérrez-Romero M., Montaño-Figueroa E.H., Martínez-Murillo C., Ramos-Peñafiel C.O. (2012). Frecuencia de leucemias agudas en un hospital de referencia. Rev. Med. Inst. Mex. Seguro Soc..

[2] Torre L.A., Bray F., Siegel R.L., Ferlay J., Lortet-Tieulent J., Jemal A. (2015). Global cancer statistics, 2012. CA Cancer J. Clin..

[3] Larson R.A. (2003). Is modulation of multidrug resistance a viable strategy for acute myeloid leukemia?. Leukemia.

[4] Caamal-Fuentes E., Torres-Tapia L.W., Simá-Polanco P., Peraza-Sánchez S.R., Moo-Puc R. (2011). Screening of plants used in Mayan traditional medicine to treat cancer-like symptoms. J. Ethnopharmacol..

[5] Valencia-Chan L.S., García-Cámara I., Torres-Tapia L.W., Moo-Puc R.E., Peraza-Sánchez S.R. (2017). Lupane-type triterpenes of *Phoradendron vernicosum*. J. Nat. Prod..

[6] Chaírez-Ramírez M.H., Moreno-Jimenez M.R., Gónzalez-Laredo R.F., Gallegos-Infante J.A., Rocha-Gúzman N.E. (2016). Lupane-type triterpenes and their anti-cancer activities against most common malignant tumors: A review. EXCLI J..

[7] Ye B., Ji Z.-N. (2012). 23-Hydroxybetulinic acid-induced HL-60 cell autophagic apoptosis and its molecular mechanism. Nat. Prod. Res..

[8] Raghuvar Gopal D.V., Narkar A.A., Badrinath Y., Mishra K.P., Joshi D.S. (2005). Betulinic acid induces apoptosis in human chronic myelogenous leukemia (CML) cell line K-562 without altering the levels of Bcr-Abl. Toxicol. Lett..

[9] Wu Q., He J., Fang J., Hong M. (2010). Antitumor effect of betulinic acid on human acute leukemia K562 cell in vitro. J. Huazhong Univ Sci. Technol. Med. Sci..

[10] Erhardt H., Fulda S., Führer M., Debatin K.M., Jeremias I. (2004). Betulinic acid induced apoptosis in leukemia cells. Leukemia.

[11] Dash S.K., Chattopadhyay S., Dash S.S., Tripathy S., Das B., Mahapatra S.K., Bag B.G., Karmakar P., Roy S. (2015). Self assembled nano fibers of betulinic acid: A selective inducer for ROS/TNF-alpha pathway mediated leukemic cell death. Bioorg. Chem..

[12] Barreto-Vianna D.R., Gotardi J., Baggio Gnoatto S.C., Pilger D.A. (2021). Natural and semisynthetic pentacyclic triterpenes for chronic myeloid leukemia therapy: Reality, challenges and perspectives. ChemMedChem.

[13] Lipinski C.A., Lombardo F., Dominy B.W., Feeney P.J. (2001). Experimental and computational approaches to estimate solubility and permeability in drug discovery and development settings. Adv. Drug Deliv. Rev..

[14] Daina A., Michielin O., Zoete V. (2017). SwissADME: A free web tool to evaluate pharmacokinetics, druglikeness and medicinal chemistry friendliness of small molecules. Sci. Rep..

[15] Ghosh S.K., Dhungana K., Headley A.D., Ni B. (2012). Highly enantioselective and recyclable organocatalytic Michael addition of malonates to α,β-unsaturated aldehydes in aqueous media. Org. Biomol. Chem..

[16] Hata K., Ogawa S., Makino M., Mukaiyama T., Hori K., Iida T., Fujimoto Y. (2008). Lupane triterpenes with a carbonyl group at C-20 induce cancer cell apoptosis. J. Nat. Med..

[17] Hata K., Hori K., Ogasawara H., Takihashi S. (2003). Anti-leukemia activities of lup-28-al-20(29)-en-3-one, a lupane triterpene. Toxicol. Lett..

[18] Mutai C., Abatis D., Vagias C., Moreau D., Roussakis C., Roussis V. (2007). Lupane triterpenoids from *Acacia mellifera* with cytotoxic activity. Molecules.

[19] Mutai C., Abatis D., Vagias C., Moreau D., Roussakis C., Roussis V. (2004). Cytotoxic lupane-type triterpenoids from *Acacia mellifera*. Phytochemistry.

[20] Nakayachi T., Yasumoto E., Nakano K., Morshed S.R., Hashimoto K., Kikuchi H., Nishikawa H., Kawase M., Sakagami H. (2004). Structure-activity relationships of α,β-unsaturated ketones as assessed by their cytotoxicity against oral tumor cells. Anticancer Res..

[21] Hossian M., Das U., Dimmock J.R. (2019). Recent advances in α,β-unsaturated carbonyl compounds as mitochondrial toxins. Eur. J. Med. Chem..

[22] Moreno-Lorenzana D.L., Avilés-Vazquez S., Sandoval-Esquivel M.A., Alvarado-Moreno A., Ortiz-Navarrete V., Torres-Martínez H., Ayala-Sánchez M., Mayani H., Chavez-González A. (2016). CDKIs p18INK4c and p57Kip2 are involved in quiescence of CML leukemic stem cells after treatment with TKI. Cell Cycle.

[23] Milojkovic D., Apperley J. (2009). Mechanisms of resistance to imatinib and second-generation tyrosine inhibitors in chronic myeloid leukemia. Clin. Cancer Res..

[24] Apperley J. (2007). Part I: Mechanisms of resistance to imatinib in chronic myeloid leukemia. Lancet Oncol..

[25] Sánchez-Martínez C., Gelbert L.M., Lallena M.J., de Dios A. (2015). Cyclin dependent kinase (CDK) inhibitors as anticancer drugs. Bioorg. Med. Chem. Lett..

[26] Sedlacek H.H. (2001). Mechanisms of action of flavopiridol. Crit. Rev. Oncol. Hematol..

[27] Rea D., Legros L., Raffaoux E., Thomas X., Turlure P., Maury S., Dupriez B., Pigneux A., Choufi B., Reman O. (2006). High-dose imatinib mesylate combined with vincristine and dexamethasone (DIV regimen) as induction therapy in patients with resistant Philadelphia-positive acute lymphoblastic leukemia and lymphoid blast crisis of chronic myeloid leukemia. Leukemia.

[28] Pishvaian M.J., Slack R., Koh E.Y., Baumer J.H., Hartley M., Cotaria I., Deeken J., He A.R., Hwang J., Malik S. (2012). A phase I clinical trial of the combination of imatinib and paclitaxel in patients with advanced or metastatic solid tumors refractory to standard therapy. Cancer Chemother. Pharmacol..

[29] Kiyoi H., Kawashima N., Ishikawa Y. (2020). *FLT3* mutations in acute myeloid leukemia: Therapeutic paradigm beyond inhibitor development. Cancer Sci..

[30] He R., Liu B., Yang C.R., Han Z.C. (2003). Inhibition of K562 leukemia angiogenesis and growth by expression of antisense vascular endothelial growth factor (VEGF) sequence. Cancer Gene Ther..

[31] Song G., Li Y., Jaing G. (2012). Role of VEGF/VEGFR in the pathogenesis of leukemias and as treatment targets (review). Oncol. Rep..

[32] Bueno-da-Silva A., Brumatti G., Russo F., Green D.R., Amarante-Mendes G.P. (2003). Bcr-Abl-mediated resistance to apoptosis is independent of constant tyrosine-kinase activity. Cell Death Differ..

[33] Daina A., Zoete V. (2016). A boiled-egg to predict gastrointestinal absorption and brain penetration of small molecules. ChemMedChem.

[34] Newby D., Freitas A.A., Ghafourain T. (2015). Decision trees to characterize the roles of permeability and solubility on the prediction of oral absorption. Eur. J. Med. Chem..

[35] Deodhar M., Al-Rihani S.B., Arwood M.J., Darakjian L., Dow P., Turgeon J., Michaud V. (2020). Mechanisms of CYP450 inhibition: Understanding drug-drug interactions due to mechanism-based inhibition in clinical practice. Pharmaceutics.

[36] Mlala S., Oyedeji A.O., Gondwe M., Oyedeji O.O. (2019). Ursolic acid and its derivatives as bioactive agents. Molecules.

[37] Milan A., Mioc A., Prodea A., Mioc M., Buzatu R., Ghiulai R., Racoviceanu R., Caruntu F., Şoica C. (2022). The optimized delivery of triterpenes by liposomal nanoformulations: Overcoming the challenges. Int. J. Mol. Sci..

[38] Khwaza V., Mlala S., Oyedeji O.O., Aderibigbe B.A. (2021). Pentacyclic triterpenoids with nitrogen-containing heterocyclic moiety, privileged hybrids in anticancer drug discovery. Molecules.

[39] Bachořík J., Urban M. (2021). Biocatalysis in the chemistry of lupane triterpenoids. Molecules.

[40] Sidova V., Zoufaly P., Pokorny J., Dzubak P., Hajduch M., Popa I., Urban M. (2017). Cytotoxic conjugates of betulinic acid and substituted triazoles prepared by Huisgen Cycloaddition from 30-azidoderivatives. PLoS ONE.

[41] Dubinin M.V., Semenova A.A., Nedopekina D.A., Davletshin E.V., Spivak A.Y., Belosludtsev K.N. (2021). Effect of F16-Betulin conjugate on mitochondrial membranes and its role in cell death initiation. Membranes.

[42] Spivak A.Y., Nedopekina D.A., Gubaidullin R.R., Davetshin E.V., Tukhbatullin A.A., D’yakonov V.A., Yunusbaeva M.M., Dzhemileva L.U., Dzhemilev U.M. (2021). Pentacyclic triterpene acid conjugated with mitochondria-targeting cation F16: Synthesis and evaluation of cytotoxic activities. Med. Chem. Res..

[43] Karpova M.B., Sanmun D., Henter J.I., Fadeel B. (2006). Betulinic acid, a natural cytotoxic agents, fails to trigger apoptosis in human Burkitt’s lymphoma-derived B-cell lines. Int. J. Cancer.

[44] Soymaya T., Lakshmipriya T., Klika K.D., Jayasree P.R., Kumar P.R.M. (2021). Anticancer potential of rhizome extract and a labdane diterpenoid from *Curcuma mutabilis* plant endemic to Western Ghats of India. Sci. Rep..

[45] RCSB PDB, Protein Data Bank. https://www.rcsb.org.

[46] Zhao H., Caflisch A. (2013). Discovery of ZAP70 inhibitors by high-throughput docking into a conformation of its kinase domain generated by molecular dynamics. Bioorg. Med. Chem. Lett..

[47] Kalinichenko E., Faryna A., Kondrateva V., Vlasova A., Shevchenko V., Melnik A., Avdoshko O., Belko A. (2019). Synthesis, biological activities and docking studies of novel 4-(arylaminomethyl)benzamide derivatives as potential tyrosine kinase inhibitors. Molecules.

[48] Fu L., Mou J., Deng Y., Ren X., Qiu S. (2022). Design, synthesis, and activity assays of cyclin-dependent kinase 1 inhibitors with flavone scaffolds. Front. Chem..

[49] Gokhale P., Chauhan A., Arora A., Khandekar N., Nayarisseri A., Singh S. (2019). FLT3 inhibitor design using molecular docking based virtual screening for acute myeloid leukemia. Bioinformation.

[50] Yan T., Bai L., Zhu H., Zhang W., Lv P. (2017). Synthesis and biological evaluation of glycyrrhetic acid derivatives as potential VEGFR2 inhibitors. ChemMedChem.

[51] Yin Y., Sha S., Wang Y., Wu X., Wang S., Qiao F., Lv P., Zhu H. (2015). Discovery of new 4-alkoxyquinazoline-based derivatives as potent VEGFR2 inhibitors. Chem. Biol. Drug Des..

[52] Kajal K., Panda A., Bhat J., Chakraborty D., Bose S., Bhattacharjee P., Sarkar T., Chatterjee S., Kar S., Sa G. (2019). Andrographolide binds to ATP-binding pocket of VEGFR2 to impede VEGFA-mediated tumor-angiogenesis. Sci. Rep..

[53] Cuartas V., Aragón-Muriel A., Liscano Y., Polo-Cerón D., Crespo-Ortiz M., Quiroga J., Abonia R., Insuasty B. (2021). Anticancer activity of pyrimidodiazepines based on 2-chloro-4-anilinoquinazoline: Synthesis, DNA binding and molecular docking. RSC Adv..

