# Supplementary material for: Apoptotic and Cell Cycle Effects of Triterpenes Isolated from Phoradendron wattii on Leukemia Cell Lines
Source: Molecules. 2022 Aug 31;27(17):5616. doi: 10.3390/molecules27175616 (PMC9458143; doi:10.3390/molecules27175616)
Supplement: Supplementary file 1 [file molecules-27-05616-s001.zip › molecules-1880860-supplementary.pdf]

Apoptotic and Cell Cycle Effects of Triterpenes Isolated from *Phoradendron wattii* on Leukemia Cell Lines

Lía S. Valencia-Chan [1], Dafné Moreno-Lorenzana [2,3], Jimmy Josué Ceballos-Cruz [4], Sergio R. Peraza-Sánchez [4], Antonieta Chávez-González [2] and Rosa E. Moo-Puc [1,*]

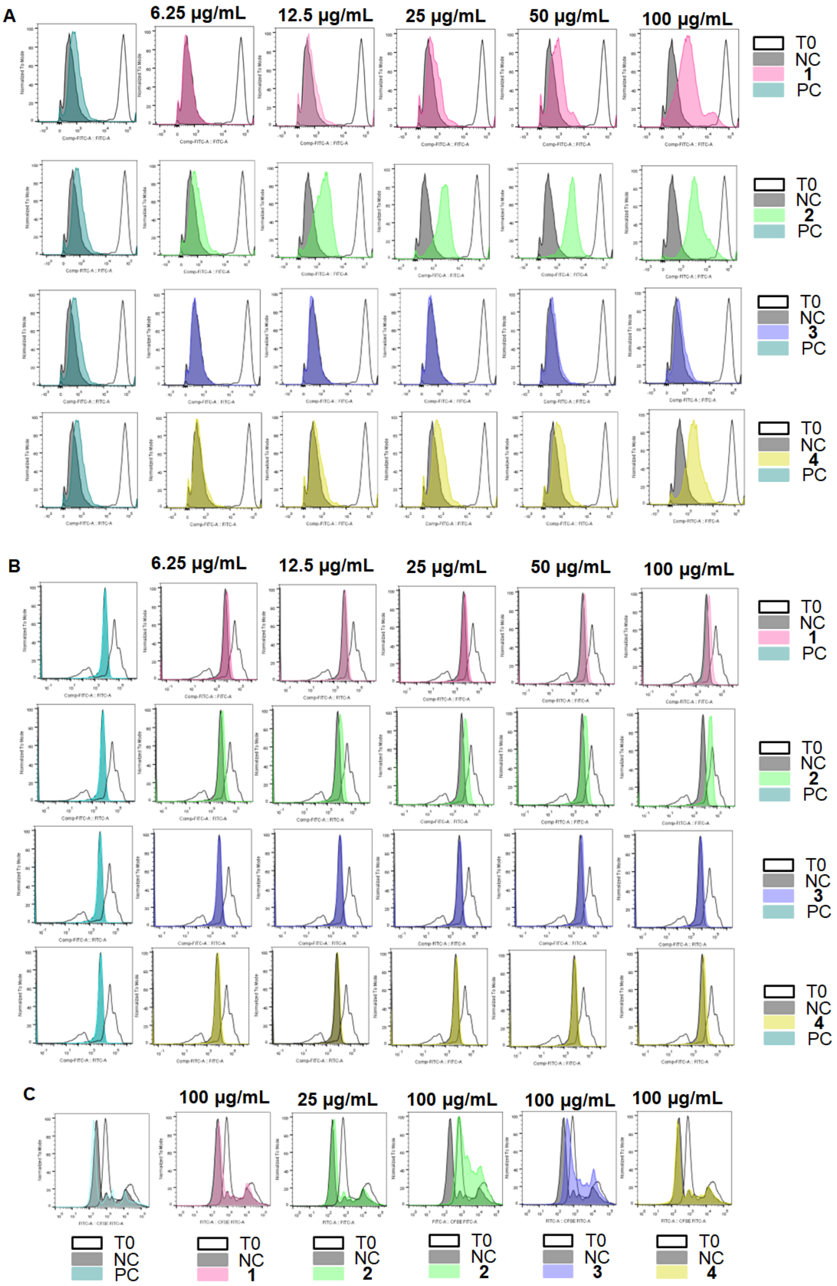

Figure S1. Effect of different concentrations of compounds **1–4** in cell proliferation. K562 (**A**), HL60 (**B**) cell lines and normal MNC (**C**) were cultured 48 h in presence of different concentrations of compounds **1–4** and compared with their correspondent CFSE content at the beginning of culture (control T0).

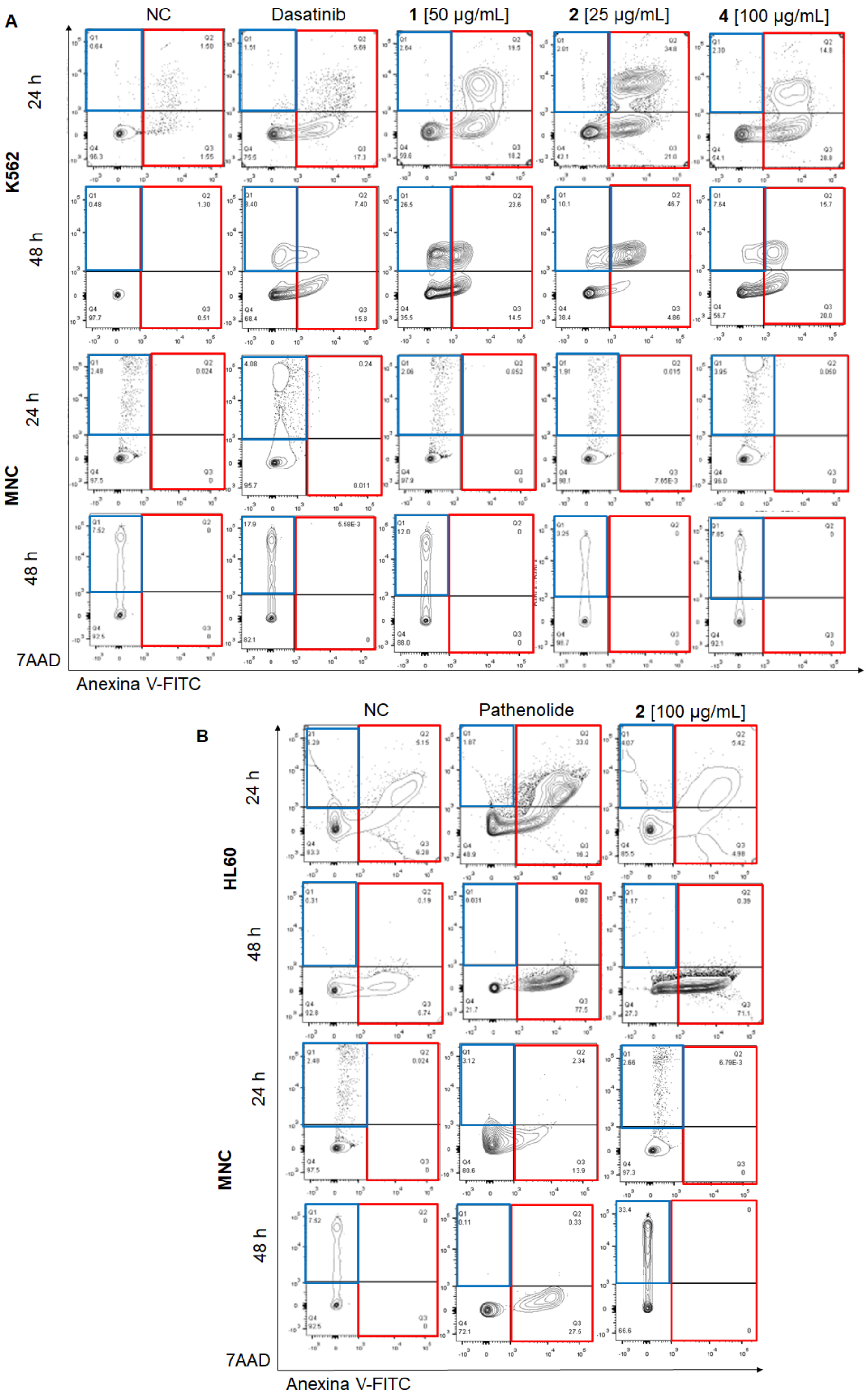

Figure S2. Effect of different compounds in cell apoptosis death. K562 (**A**), HL60 (**B**) cell lines and normal MNC (**C**) were cultured 24 and 48 h in presence of different concentrations of compounds. The population of apoptotic cells (positive annexin V-FITC, marked in red box) and necrosis (single positive 7-AAD, marked in blue box) pathway.

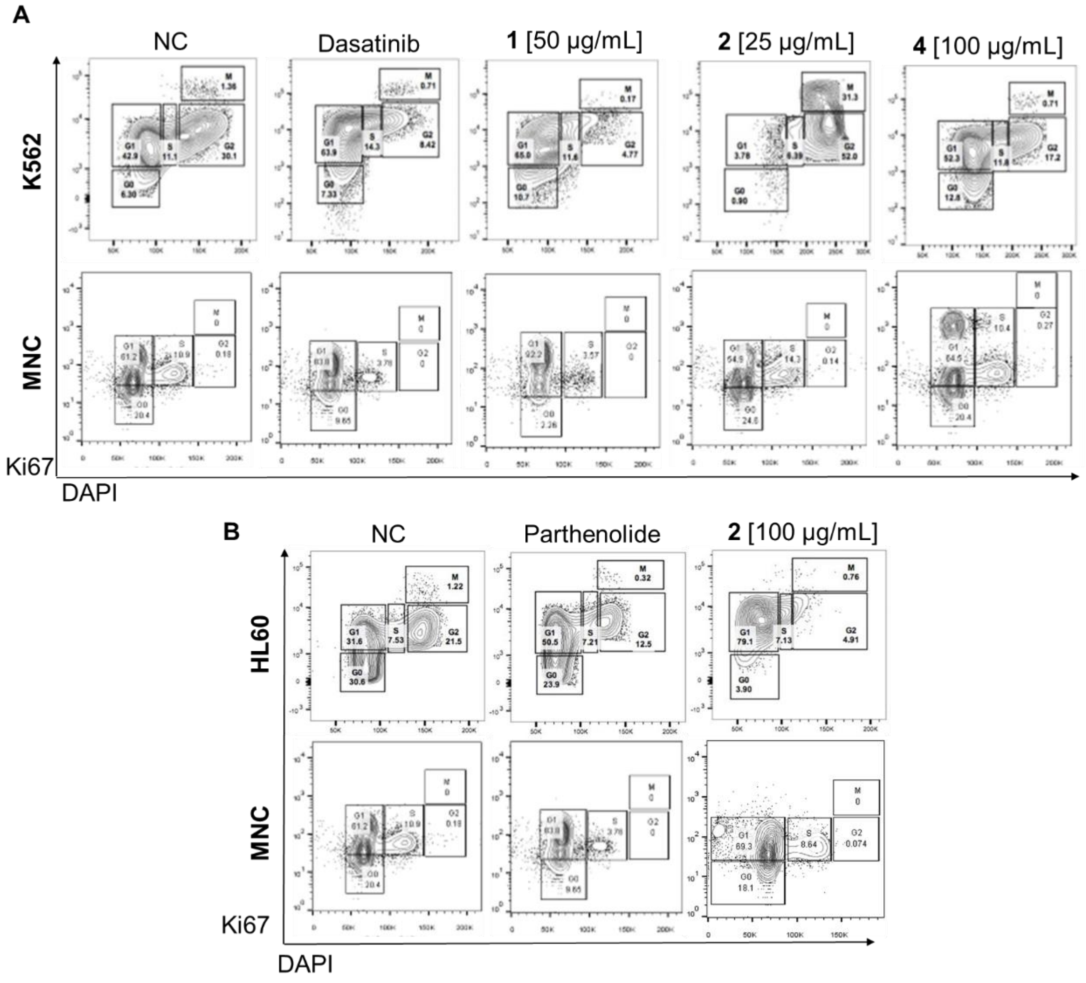

Figure S3. Effect of different compounds on the cell cycle status. K562 (**A**), HL60 (**B**) cell lines and normal NMC were cultured 48 h in presence of different concentrations of compounds to analyze the cell cycle status using Ki67 and DAPI staining. Representative doth plot is showing and graphics represent the average of three replicates comparing each cell line with the corresponding normal MNC.

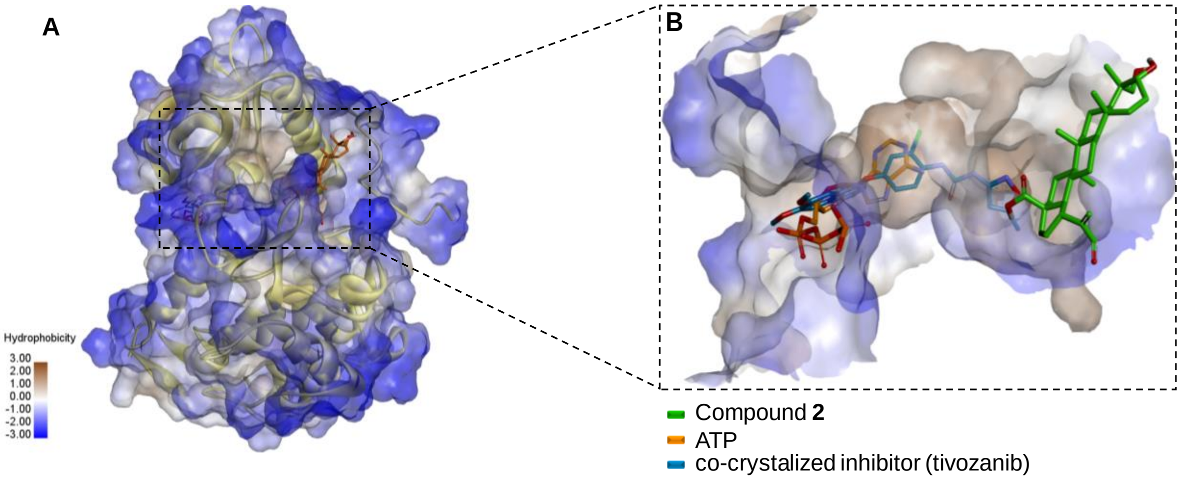

Figure S4. Binding modes of the studied compounds at the VEGFR binding site. (**A**) Binding site of tivozanib, ATP and compound **2**, (**B**) Close-up of the binding site of tivozanib (Re-docking: 0.286 Å), ATP and compound **2**.

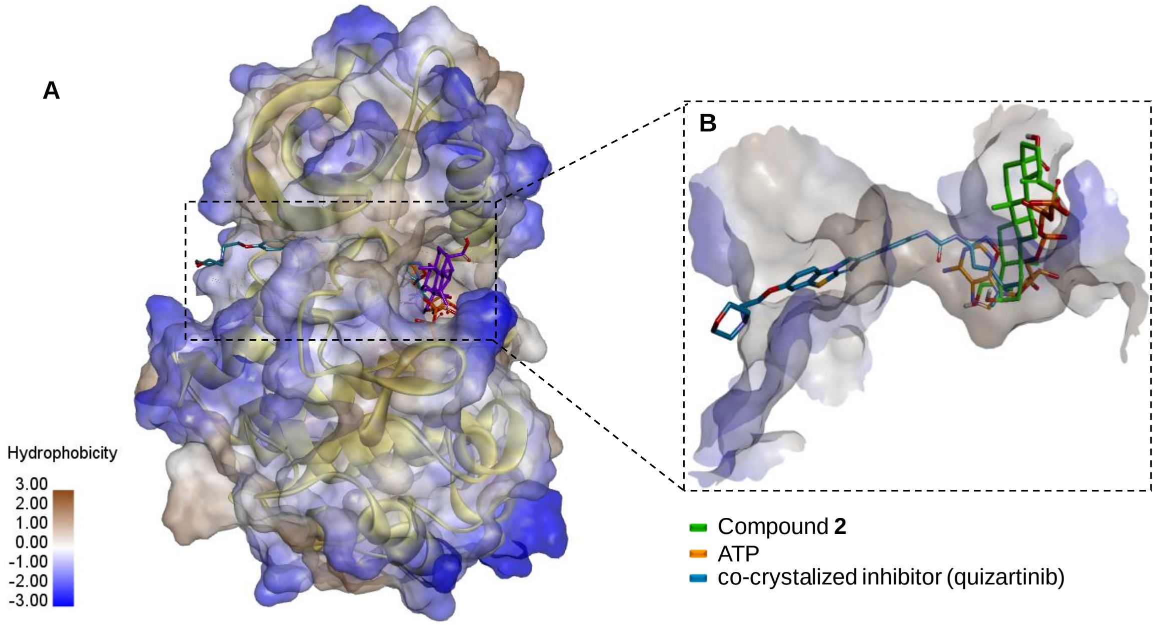

Figure S5. Binding modes of studied compounds at the FLT3 binding site. (**A**) Binding site of quizartinib, ATP and compound **2**, (**B**) Close-up of the binding site of quizartinib (Re-docking: 1.472 Å), ATP and compound **2**.

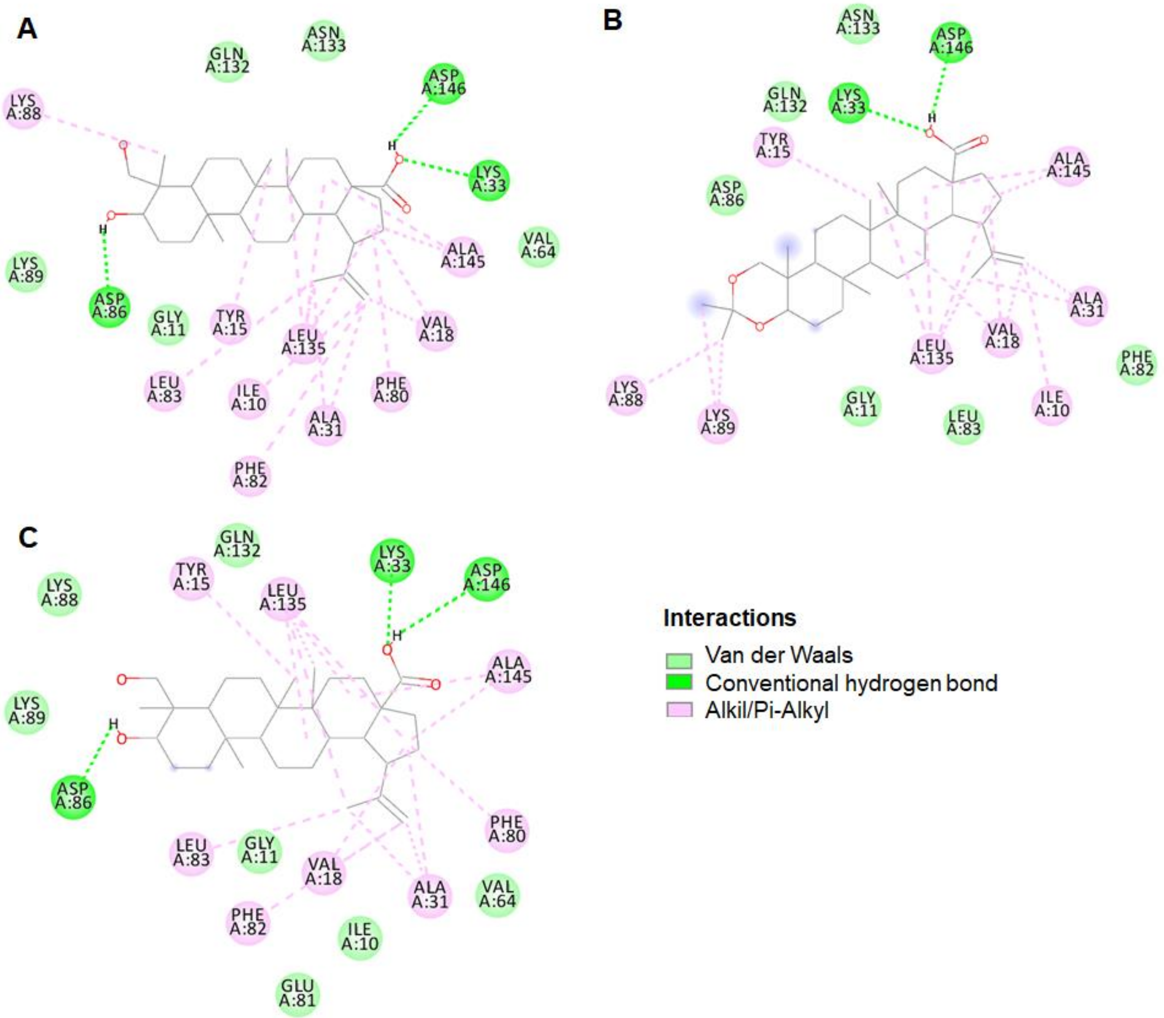

Figure S6. Interactions of the compounds **1**, **3**, and **4** at CDK1 binding site. (**A**) Visualization of interactions between compound **1** inside CDK1 binding site, (**B**) Visualization of interactions between compound **3** inside CDK1 binding site, (**C**) Visualization of interactions between compound **4** inside CDK1 binding site.
